# Targeting Lifestyle Behavior Change in Adults with NAFLD During a 20-min Consultation: Summary of the Dietary and Exercise Literature

**DOI:** 10.1007/s11894-016-0485-1

**Published:** 2016-02-23

**Authors:** Kate Hallsworth, Leah Avery, Michael I. Trenell

**Affiliations:** Institute of Cellular Medicine, Newcastle University, MoveLab, 4th Floor William Leech Building, Newcastle upon Tyne, NE2 4HH UK

**Keywords:** Non-alcoholic fatty liver disease (NAFLD), Behavior change, Weight loss, Diet, Exercise, Physical activity

## Abstract

Non-alcoholic fatty liver disease (NAFLD) is largely linked to poor diet, lack of physical activity/exercise, and being overweight. In the absence of approved pharmaceutical agents, lifestyle modification, encompassing dietary change and increased physical activity/exercise to initiate weight loss, is the recommended therapy for NAFLD. Despite this, the use of lifestyle therapy within clinical settings is lacking with limited guidance available about what it should involve, how it should be delivered, and whether it can be feasibly delivered as part of standard care. This paper highlights the evidence for the use of lifestyle modification in NAFLD. While there is evidence to support use of behavioral strategies to support lifestyle behavior change in other clinical populations, these are yet to be assessed in people with NAFLD. However, there is sufficient evidence to suggest that behavioral intervention targeting diet and physical activity to promote weight loss in general is effective and a number of practical strategies are presented on how this could be achieved.

## Introduction

Non-alcoholic fatty liver disease (NAFLD) is rapidly becoming the most common liver condition in the world and is largely linked to increased calorie consumption/poor diet, lack of physical activity/exercise, and being overweight. NAFLD represents a spectrum of liver disease ranging from isolated fatty liver to progressive non-alcoholic steatohepatitis (NASH), hepatic fibrosis, and cirrhosis. The overall prevalence of NAFLD in adults in Western countries is estimated to be 20–33 % [1, 2], and incidence increases in people with obesity and type 2 diabetes (T2DM). Patients with simple fatty liver have a relatively “benign” liver prognosis with a 1–2 % risk of developing evidence of cirrhosis over 15–20 years [3], while up to 5–11 % of those with NASH develop end-stage liver disease [4•]. Furthermore, excess liver fat markedly increases cardiovascular disease risk, and heart disease is the second most common cause of death in those with NAFLD [5].

In the absence of approved pharmaceutical agents, lifestyle modification, involving weight loss, initiated by dietary and physical activity/exercise behavior change, is the primary recommended therapy for NAFLD [6]. Despite this, the use of lifestyle therapies in the clinical setting is severely lacking with limited guidance about how this should be delivered and what should be included as part of standard care. The American Association for the Study of Liver Disease published practice guidelines in 2012 regarding the diagnosis and management of NAFLD [7]; however, these were vague, particularly with regard to targeting lifestyle behavior change as a treatment modality for NAFLD. The Lancet Standing Commission [8] recommends the promotion of healthy lifestyles to address the current obesity epidemic in the fight against NAFLD but again fails to provide specific details as to how this should or could be achieved.

Evidence supporting the use of lifestyle therapy is strong and has shown significant reductions in liver fat and improvements in glucose control/insulin sensitivity in those with NAFLD after short dietary and exercise interventions [9•]. The majority of research assessing the effect of lifestyle modification on NAFLD has been in the early stages of the disease (i.e., in people with simple steatosis) rather than the later stages. Lifestyle interventions designed to induce weight loss through reduced energy intake (dietary behavior change only) in NAFLD have led to reductions in liver fat of 42–81 % [10–12] with the greatest reductions in liver fat observed in people who lose the most weight. Weight loss has also demonstrated to be successful in treating NASH, again with a greater weight reduction associated with better improvements in histology on repeat biopsy [13]. However, weight loss is difficult to achieve in the clinical setting, and weight loss maintenance poses a significant challenge; thus, an alternative approach is required. A few small-scale studies have investigated the effect of exercise alone (without weight loss or dietary change) in NAFLD. These studies have consistently shown a reduction in liver fat of 12–27 % and/or improvements in liver enzymes following short programs of traditional aerobic [14, 15], modified high-intensity interval training [16], or resistance exercise [17]. Studies investigating the combined effects of diet and exercise in NAFLD have reported reductions in liver fat of 23–51 % [18, 19], and one study reported significant changes in NAFLD histology on repeat biopsy [20]. These studies provide support for the use of lifestyle modification incorporating diet and exercise, with weight loss offering the largest improvements for liver health.

A weight loss goal of circa 10 % has been recommended for patients with NAFLD [13, 21, 22]; however, weight loss is difficult to achieve and sustain even in well-conducted and resourced clinical trials. If patients can lose a small amount of weight through diet and/or exercise, they can reduce their risk of disease progression and worsening metabolic control [9]. Patients electing to exercise who remain weight neutral can benefit from an improvement in body composition with an increase in lean-mass to fat-mass ratio and thus have a greater fat burning capacity and less fatty tissue despite lack of weight loss [6, 7]. Weight loss is a key in NAFLD; however, if a patient is unable to lose weight via dietary behavior change alone, physical activity and exercise offer an alternative route to improving liver and metabolic health.

Public health guidelines recommend that adults undertake a minimum of 150 min/week of moderate-vigorous leisure-time physical activity to decrease risk of metabolic disease [23, 24]. However, the majority of people in the general population do not strive for or achieve this recommendation, and this is likely contributing to increased prevalence of obesity, T2DM, and NAFLD. Low levels of physical activity are associated with an increased risk of developing chronic diseases, including cardiovascular disease [25], T2DM [26, 27], and cancer [28]. Interventions that increase physical activity have shown to be effective in the prevention of these diseases [29–33] but have yet to be assessed in NAFLD. Research has shown that objectively measured levels of physical activity behavior are lower in people with NAFLD than in matched healthy controls and that people with NAFLD are more sedentary [34•]. Furthermore, physical activity levels are inversely related to liver fat [35], and as such people with NAFLD should be encouraged to increase their physical activity levels. This may be in the form of increasing day-to-day physical activity (e.g., walking) or by taking part in structured exercise programs.

### Key Points from Recent Dietary Studies

Studies investigating dietary composition have been largely inconclusive regarding which type of diet is the most effective for managing NAFLD [9•, 21, 36]. The key findings point towards calorie reduction as being the most important factor in facilitating weight loss regardless of the type of diet a person consumes. Low calorie diets and very low calorie diets (including meal replacement products) are effective for inducing rapid weight loss [36]; however, patients are likely to require a greater level of support to adhere to these diets and would require close monitoring, particularly if they have diabetes. Diets enriched with fructose, trans-fatty acids and saturated fat (i.e., the typical western diet) have shown associations with the development of NAFLD [37, 38]. Therefore, restricting calories from these common sources may be important, particularly considering higher fructose consumption has also been associated with an increase in fibrosis severity in patients with NAFLD [39]. For long-term weight loss maintenance, a general healthy balanced diet is recommended [6], although patients will also require specific personalized information about energy intake and expenditure as well as support to better self-regulate their diet and physical activity levels.

### Key Points from Recent Physical Activity/Exercise Studies

Patients with NAFLD have been shown to undertake lower levels of physical activity and are more sedentary than their healthy counterparts [34•, 40]. Increasing physical activity levels and reducing sedentary time not only increases energy expenditure, thus enhancing weight loss, but also has beneficial effects on metabolic and cardiac risks [16, 41]. Exercise studies have shown improvements in NAFLD in the absence of weight loss and evidence supports different types (aerobic, resistance and high-intensity interval training [12–16]) and doses of exercise as being effective [42•, 43]. This is good news for patients because it provides the necessary evidence that any form of exercise is likely to be beneficial and so provides patient choice. There is strong evidence to support the use of pedometers and activity trackers to provide estimation of baseline activity levels and for use as motivational and volitional tools to increase physical activity behavior and improve health [44]. Central to physical activity/exercise promotion is supporting patients to make an initial step towards increasing their physical activity levels. This involves assisting them to identify salient reasons for making a change to physical activity levels, selecting an activity of choice, advising them on the necessary amount of activity required to attain a pre-set end goal (e.g., reduced liver fat), providing feedback on performance in relation to clinical outcomes, and providing ongoing support to prevent relapse. The need for patients to find an activity they enjoy is paramount to increase the likelihood of long-term sustainable changes. Supporting patients to identify ways in which they can increase their everyday level of physical activity or helping them to locate exercise schemes in the community can be useful to support this.

### Clinical Observations: Targeting Lifestyle Behavior Change of Adults with NAFLD

Typically, patients with NAFLD present to clinicians as overweight or obese with a host of co-morbidities likely to include overt T2DM and/or CVD or risk factors for the development of both diseases. Patients are largely inactive and report difficulty losing weight and maintaining weight loss. The challenge with these patients is to motivate them to want to make long-term changes to their lifestyle behaviors and to support them to make changes that will result in weight loss and weight loss maintenance and thus benefit their liver, metabolic, and cardiac health [9]. Clinical care needs to be “patient-centered” with less of a didactic approach to the consultation and more of a two-way conversation where the clinician attempts to understand the patients’ previous experiences with a view to moving forward with treatment.

The first challenge is to ensure that patients understand their diagnosis: what NAFLD is, why they have developed it, and what they can do to halt disease progression or reverse their condition. It is also important from the outset to explain to the patient that their liver disease is linked to lifestyle behaviors (i.e., diet and physical activity levels) and excess weight as opposed to alcohol consumption in the majority of cases. Many patients link liver disease solely to excess drinking, and this can be a significant barrier to acceptance of their diagnosis, particularly if their alcohol consumption is within the recommended limits. This can create difficulties when trying to move a patient forward with treatment. Traditionally, patients with NAFLD are told/advised by clinicians to lose weight; however, commonly, they are not supported to make any meaningful changes to their lifestyle behaviors. This frequently results in unsuccessful weight loss attempts and disengagement with services. Often, these are people that have tried (and failed) a multitude of different diets over several years and have received conflicting information from health-care professionals, friends, family members, and the media, which has resulted in confusion as to what the correct advice is.

To promote successful lifestyle behavior change, greater emphasis is being placed on the use of evidence-informed behavior change techniques [45] and/or brief motivational techniques [46] for use within clinical settings. These techniques are used to increase motivation (i.e., address common misconceptions, identify personal reasons for change and ways in which patients can successfully change their lifestyle behaviors to increase intention) and support volition (i.e., promotion of practical strategies that patients can use such as goal setting and barrier identification to plan behavioral changes and maintain changes over time) [47•, 48–50]. Previous experiences of successful/unsuccessful lifestyle behavior changes can be explored to identify what worked well so that these experiences can be utilized to inform future behavioral change strategies.

At the start of the management process, it is important to determine what a patient already knows about NAFLD, why they believe they have developed the disease, and whether they have been advised of ways in which they can manage it (i.e., the very first consultation may involve addressing misconceptions, however by providing patients with the correct information; this in itself can raise self-efficacy and make lifestyle behavior change more likely). Once it has been established that the patient is ready and willing to proceed with lifestyle behavior changes, it is important to explore and understand their current diet and eating habits, for example, what type of foods do they eat, the size of their portions, learning who does the cooking/food shopping in the household, have they already started to think about or make any changes, and have these been successful? It may be useful for the patient to use a food diary to record their current dietary intake in detail. This not only gives a baseline measure to facilitate feedback provision but can also be used to identify any patterns in behavior, track any future changes, and provides a good opportunity to start a discussion around making changes to the diet. If a patient is unable to provide a good indicator of their portion sizes of different food types, they can take a photograph of their meal on their plate that will help to “visually quantify” how much they are eating. Most patients will need to reduce their portion sizes of some, if not all, food types and buying a smaller plate on which to eat their meals is often a good way of doing this without their “normal” plate appearing empty. As discussed previously, the evidence does not support a particular type of diet for weight loss in NAFLD; the emphasis currently is on the reduction of calorie consumption.

Physical activity and exercise are often overlooked during the clinical consultation in patients with NAFLD. This is despite evidence showing the positive benefits on liver, metabolic, and cardiac health. It is beneficial to obtain a baseline measure of physical activity for each patient, including the types of activities they engage with to better understand their capabilities and the intensity at which they are working at. This can be completed via an activity diary or ideally using an objective measure which captures all incidental and planned activity over a 7-day period. Pedometers are a relatively inexpensive tool to capture step-count throughout the day but do not provide any information on activity duration, intensity, or non-stepping activities. Furthermore, they do not provide any context or information about the actual activities undertaken. Activity monitors can provide added information with regard to energy expenditure, frequency, duration, and intensity of activities performed and data on daily activity profile that is useful to target sedentary behavior. These monitors are more expensive and are mainly used for research purposes; however, with the increased use of smart phones, patients can often use Apps to track their activity in a more sophisticated way than using a traditional pedometer. Some Apps will use global positioning system (GPS) technology and allow a patient to track their walking, not only in terms of step count but also distance and speed. Having an objective measure of baseline activity levels allows clinicians to work with patients to set appropriate and realistic goals. For example, if a patient is currently achieving 2000 steps per day, it is unlikely that they will reach the recommended target of 10,000 steps per day [23] immediately; therefore, graded behavioral goal setting may be more appropriate, although this should be explored and discussed with the patient (i.e., an assumption should not be made). Supporting patients to set walking goals may be an appropriate starting point to increase their physical activity levels as opposed to a structured exercise program. Conversely, someone that has co-morbidities that restrict their walking may benefit from sign-posting to a swimming group or chair-based exercise program. The key to long-term success in supporting patients to increase their physical activity/exercise levels is to establish what motivates them to make lifestyle behavior changes and what types of activities are in line with their capabilities and preferences. Once this is established, patients can be sign-posted to activities of their choice or link with other services that may offer these opportunities. To facilitate such a process, it is beneficial to have an up-to-date list of local physical activity/exercise providers to ensure the information is available at a time when the patient requests it (i.e., at a time where they are motivated or a key teachable moment). Maintaining close relationships with local leisure service teams can prove invaluable as they can help to support patients with NAFLD to make and sustain these lifestyle behavior changes.

**Fig. 1:**
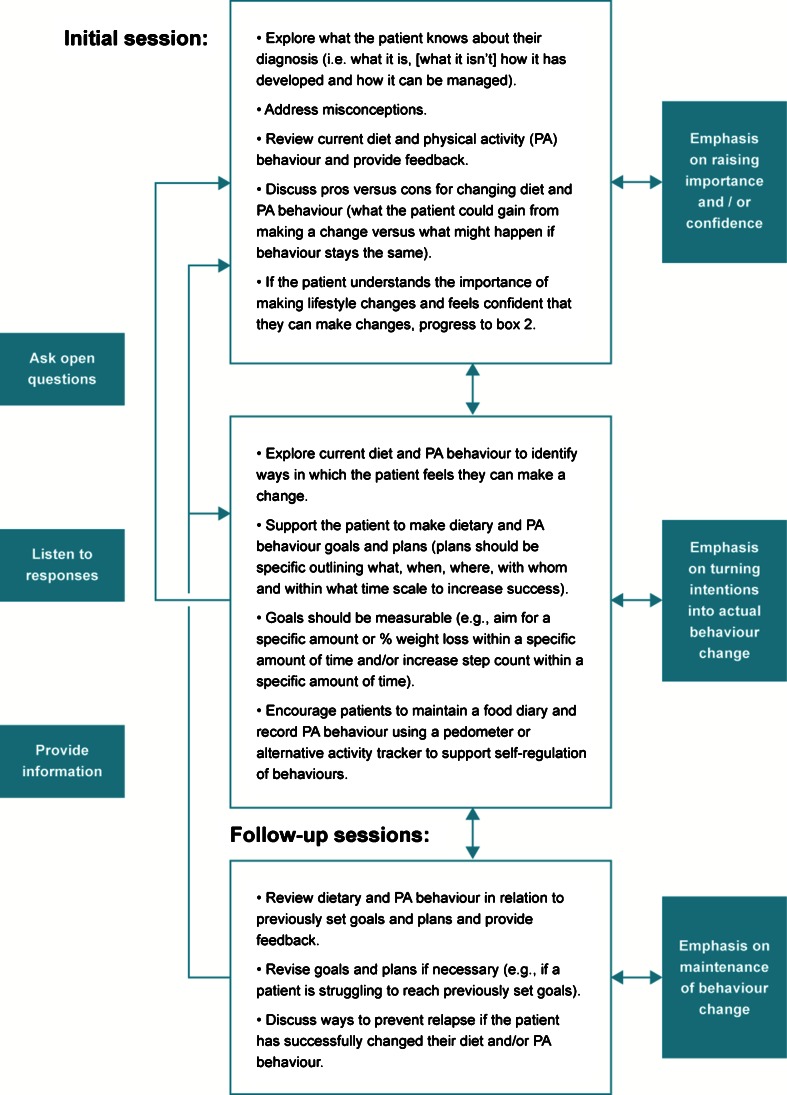
Flow diagram outlining the process for targeting lifestyle behavior change during NAFLD consultations

As referred to previously, people with NAFLD rarely receive support to make meaningful and sustainable changes to diet and physical activity behavior. This is potentially due to a number of factors including lack of knowledge and awareness on the part of the clinician of the importance of lifestyle factors in the context of NAFLD. However, the most significant factor is likely to be a lack of training in the delivery of behavior change interventions (i.e., use of behavior change and brief motivational techniques). When targeting lifestyle behavior change, we can typically think of the process occurring in three stages. The first involves determining whether the patient regards changing their diet and/or physical activity behavior as important (i.e., whether they believe changing behavior will make a difference) and whether they feel confident that they can make changes that will make a difference (i.e., whether they feel practically equipped to make changes and whether self-efficacy is high). If importance and confidence levels are high, then the patient is likely to feel motivated to move forward and make goals and plans. If not, the clinician should work with the patient to explore attitudes, beliefs, and self-efficacy first to increase the importance of making lifestyle behavior changes and to enhance self-efficacy. The second stage involves working with patients to make realistic goals and detailed plans that they feel are sustainable and sensitive to their everyday lives. These goals and plans should be capable of initiating a change in the target behavior(s) if they have been developed in collaboration with the patient. Self-monitoring at this stage can help to self-regulate lifestyle behaviors and increases self-efficacy when the patient starts to attain their goals. The final stage involves equipping patients with practical strategies such as methods to pre-plan ways to overcome barriers to prevent relapse and self-monitoring behavior to positively reinforce any changes made (i.e., increase self-efficacy). See Fig. 1.

There are a range of behavior change techniques and behavior change counseling techniques available for use, some with a stronger evidence base than others for targeting each stage in the process. For example, use of self-regulatory techniques such as self-monitoring has a good evidence base, and when paired with problem-solving and coping planning is regularly effective for changing physical activity behavior [51, 52]. However, there is currently a pressing need for training provision in health behavior change for clinicians to target lifestyle behaviors in the context of NAFLD.

## Conclusions

Targeting lifestyle modification in people with NAFLD is vitally important, not only due to the high disease prevalence but also since excess liver fat is an independent risk factor for the development of cardiovascular disease and T2DM. Reducing liver fat and its mediators should be beneficial for delaying or preventing the onset of these life-threatening conditions, which in turn reduces the burden placed on health services. Clinicians are inevitably going to be managing increasing numbers of patients with NAFLD in the future, and it is important for them to have the ability and capacity within health-care settings to motivate and support patients to make lifestyle behavior changes that can be sustained over the long term. This will require a change in the way that lifestyle interventions are delivered in the clinic, placing more emphasis on the use of behavior change strategies and engaging patients in a discussion about the choices they have in relation to their care as opposed to clinicians providing unsolicited advice. This is achievable; however, evidence-based tools and training are lacking and lifestyle behavior change interventions are likely to be a more effective solution if delivered by a multidisciplinary team.
